# Lymphatic Clearance of the Brain: Perivascular, Paravascular and Significance for Neurodegenerative Diseases

**DOI:** 10.1007/s10571-015-0273-8

**Published:** 2016-03-18

**Authors:** Erik N. T. P. Bakker, Brian J. Bacskai, Michal Arbel-Ornath, Roxana Aldea, Beatrice Bedussi, Alan W. J. Morris, Roy O. Weller, Roxana O. Carare

**Affiliations:** 1 Department of Biomedical Engineering and Physics, Academic Medical Center, 1105 AZ Amsterdam, The Netherlands; 2 Massachusetts General Hospital, Charlestown, MA 02129 USA; 3 Institute for Life Sciences, University of Southampton, Southampton, SO17 1BJ UK; 4 Faculty of Medicine, University of Southampton, Southampton General Hospital, Tremona Road, Southampton, SO16 6YD UK

**Keywords:** Perivascular, Paravascular, Interstitial fluid, Cerebrospinal fluid, Cerebrovascular basement membranes

## Abstract

The lymphatic clearance pathways of the brain are different compared to the other organs of the body and have been the subject of heated debates. Drainage of brain extracellular fluids, particularly interstitial fluid (ISF) and cerebrospinal fluid (CSF), is not only important for volume regulation, but also for removal of waste products such as amyloid beta (Aβ). CSF plays a special role in clinical medicine, as it is available for analysis of biomarkers for Alzheimer’s disease. Despite the lack of a complete anatomical and physiological picture of the communications between the subarachnoid space (SAS) and the brain parenchyma, it is often assumed that Aβ is cleared from the cerebral ISF into the CSF. Recent work suggests that clearance of the brain mainly occurs during sleep, with a specific role for peri- and para-vascular spaces as drainage pathways from the brain parenchyma. However, the direction of flow, the anatomical structures involved and the driving forces remain elusive, with partially conflicting data in literature. The presence of Aβ in the glia limitans in Alzheimer’s disease suggests a direct communication of ISF with CSF. Nonetheless, there is also the well-described pathology of cerebral amyloid angiopathy associated with the failure of perivascular drainage of Aβ. Herein, we review the role of the vasculature and the impact of vascular pathology on the peri- and para-vascular clearance pathways of the brain. The different views on the possible routes for ISF drainage of the brain are discussed in the context of pathological significance.

## Introduction

Extracellular fluid of the brain comprises cerebrospinal fluid (CSF) in the ventricles and subarachnoid space (SAS) and interstitial fluid (ISF) in the extracellular spaces (ECS) of the brain parenchyma. In humans, there are 280 ml of ISF and 140 ml CSF, of which 30 ml are in the ventricles, 80 ml in the cerebral SAS and 30 ml in the spinal SAS (Bozanovic-Sosic et al. 2001). CSF acts as a buoyancy fluid for the human brain and, considering that specific gravities of the brain and CSF are similar, CSF effectively reduces the weight of the brain in the skull. CSF is produced by active secretion from the choroid plexuses in the ventricles and is resorbed by arachnoid villi. It is however becoming clear that this traditional view on CSF circulation is too simplistic and much more complex than previously thought. The reader is referred to an excellent review on this topic by (Brinker et al. 2014). Apart from the choroid plexuses as the major anatomical feature responsible for the production of CSF, how much CSF may also be derived from ISF is uncertain (Hladky and Barrand 2014). Recent evidence suggests a role for paravascular circulation of CSF into the brain parenchyma (Iliff et al. 2012). Here we aim to discuss the recent data on CSF and ISF circulation with a focus on the role of the peri- and para-vascular routes. We subsequently discuss how the experimental evidence maps against the evidence from neuropathological studies.

### Cerebrospinal Fluid (CSF)

The mean rate of CSF production in humans is 0.3–0.4 ml/min (500–600 ml/day) and the total volume of CSF is replaced every 5–7 h (Di Chiro 1964; Milhorat 1975). The composition of CSF is determined by the local metabolism, by the restriction of intercellular diffusion and specialized transport mechanisms at the blood-CSF and blood–brain barriers and the rates of CSF production and excretion by bulk flow. Compared to plasma, the concentrations of sodium, chloride and magnesium in the CSF are higher, but the concentrations of potassium, calcium, phosphate, bicarbonate and glucose are lower. The total protein concentration of CSF is <0.5 % of that of plasma (Abbott 2004). Sodium and chloride are actively transported across the epithelial cells of the choroid plexus; water follows the concentration gradient passively. Large molecules are transported by pinocytosis from the basal to the apical surface of choroidal epithelial cells and then exocytosed into the ventricular CSF. To some extent, molecules can also be cleared from the CSF via the choroid plexus, via specific transporters that are also present at the blood–brain barrier (BBB). Examples of these are members of the ATP-binding cassette transporters and Solute Carrier families, but also peptide transporters (de Lange 2004). An important difference is that in contrast to endothelium at the BBB, the endothelium in the choroid plexus is fenestrated, resulting in highly permeable capillaries. However, as reviewed by (Damkier et al. 2013), a tight barrier is formed by the epithelial cells at the surface of the choroid plexus. Compared to the knowledge on the BBB, information about the barrier between blood and CSF is still relatively sparse.

The circulation of CSF through the lateral and third ventricles lined by ciliated cuboidal epithelium and through the aqueduct of Sylvius to the fourth ventricle and into the SAS has been reviewed recently (Brinker et al. 2014). As demonstrated by flow-sensitive MRI, CSF motion is pulsatile and related to intracranial blood vessel pulsations, resulting in a relatively small net flow of CSF from the ventricles towards the SAS (Enzmann and Pelc 1993). In humans, the SAS occupies a few millimetres over the cerebral gyri and is more extensive in the cisterns located at the base of the brain. The arachnoid layer of the meninges is composed of several layers of cells joined by desmosomes; the outer layer contains tight junctions, rendering the arachnoid impermeable to CSF (Alcolado et al. 1988).

Tracer studies in primates demonstrate that CSF drains via arachnoid villi and granulations into the blood of venous sinuses (Weed 1923). In the rat, rudimentary arachnoid villi could serve as a route of direct drainage of a small proportion of CSF into the blood. Tracers injected into the CSF flow along channels in the SAS, alongside leptomeningeal arteries, to the cribriform plate through which they enter nasal lymphatics and reach the cervical lymph nodes within 1 min (Kida et al. 1993). Ventricular injection studies show that radiolabelled insulin-like growth factor-1 is found within 1 h confined mainly to the walls of leptomeningeal arteries (Thorne et al. 2004b). The route through the cribriform plate follows the olfactory nerves (Nagra et al. 2006; Kida et al. 1993). Using Microfil as a CSF tracer, (Johnston et al. 2004) filled the subarachnoid compartment of different species, via injection into the cisterna magna. They observed a similar pathway in seven different species, from small rodents to humans, with Microfil distribution throughout the subarachnoid compartment associated with the base of the brain, in the basal cisterns in all species and also commonly in the SAS. Microfil distributed further via the cribriform plate into the lymphatic vessels of the olfactory and respiratory submucosa, underlining the presence of a lymphatic network that connects with cervical lymph nodes. Lymphatic vessels were described in 1987 in the dura mater, adjacent to the venous sinuses and draining across the cribriform plate (Andres et al. 1987). Recently, these findings have been confirmed in rodents, using fluorescent markers for lymphatic vessels and confocal microscopy that allowed the detection of these markers in relation to the dural sinuses (Aspelund et al. 2015; Louveau et al. 2015). The relative contribution of all these pathways for the drainage of CSF has not yet been established. However, for humans it has been hypothesized to be 1/3 via spinal vessels, 1/3 via the cribriform plate and 1/3 via the arachnoid granulations (Veening and Barendregt 2010).

### Interstitial Fluid (ISF)

**Fig. 1:**
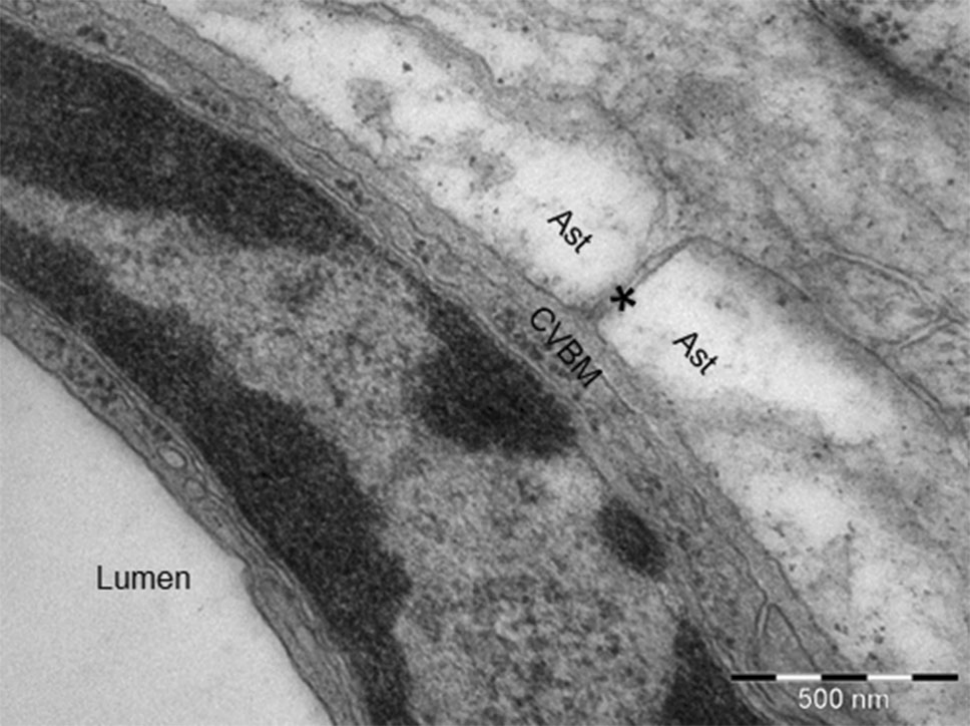
Electron micrograph showing the continuity between the extracellular spaces and the basement membrane of a cerebral capillary. The extracellular space between the astrocyte end-feet (Ast) is emphasized by a *star* and communicates directly with the cerebrovascular basement membranes (CVBM). 24-week Wiskar rat cortical capillary. Magnification: ×50,000,* scale bar* 500 nm

Electron microscopy has shown that very narrow gaps separate the cells of the nervous system (Hrabětová and Nicholson 2004; Morris et al. 2014). The gaps are interconnected and are filled with interstitial fluid; they represent the ECS that is in direct continuity with the basement membranes of capillaries (Fig. 1). The most significant exchange between blood and central nervous system (CNS) occurs at the capillary level. The cerebral capillary endothelial cells connected by tight junctions form the BBB. As recently reviewed by (Hladky and Barrand 2014), the consequence of this barrier function is that the filtration of solutes and water is considerably lower in the brain than in peripheral tissues. Formation of ISF is highly dependent on active transport of solutes across the BBB. This does allow a more controlled bidirectional exchange of molecules between the blood and the brain tissue. Nutrients, ions, oxygen, together with other fundamental molecules for CNS metabolism cross the BBB from blood towards the brain tissue. After crossing the BBB, the molecules diffuse over short distances of 8–25 μm to the surrounding cells comprising the CNS. In addition, the capillary endothelium also presents an efflux route with specific membrane transporters for the substances that the brain must clear away (e.g. Aβ, lipid soluble toxins). The movement of water mixed with the movement of solutes (e.g. Aβ) results in the flow of ISF (Abbott 2004, 2013). After entering the basement membrane at the capillary level, ISF drains along the basement membranes of cerebral arteries by bulk flow, as indicated by tracer experiments (Carare et al. 2008). Bulk flow of ISF was also reported along the white matter axon tracts (Abbott 2004). Nonetheless, the movement of ISF through the ECS surrounding the cells of nervous system depends on diffusion (Syková and Nicholson 2008; Nicholson et al. 2011). Functionally, the ECS provides a pathway for the diffusion and exchange of ions and molecules between cells.

The most widely used method for studying diffusion is the real-time iontophoretic method TMA (tetramethylammonium) (Nicholson 1992). Molecules execute “random walks” through the ECS; diffusion of solutes in the ECS is hindered by the presence of cells and their processes. The apparent diffusion coefficient ($D^{*}$) compared to the diffusion coefficient in water ($D$ *)* is measured by tortuosity ($\lambda$). Tortuosity in the ECS is a dimensionless value defined as $\lambda = (D/D^{*} )^{1/2}$; tortuosity is sensitive to factors like viscosity of the interstitial fluid and molecular size.

Due to the close apposition of cells, their processes and junctions, the hindrance (tortuosity) encountered by diffusing molecules in the grey matter is higher compared to the white matter. Furthermore, movement of molecules through the ECS is size dependent. Thus, small molecules (Mw ≤ 6600 Da) are less hindered in their passage through the brain ECS than proteins with a Mw ≥ 10,000 Da. Within different regions of the brain there are differences in the tortuosity in the grey matter; for example, the tortuosity in the hippocampus is significantly lower than that in the neocortex (Thorne et al. 2004a). In the white matter diffusion is facilitated by its structure consisting of parallel fibres with few interconnections; consequently, oedema fluid can be accommodated in the spaces between white matter fibres but not in the grey matter. Recent experimental work suggests that the dimension of the ECS during sleep increases by 60 % in mice but this still remains to be clarified and confirmed by other studies (Xie et al. 2013). These values remain unaffected by increasing age, despite that fact that ageing results in profound morphological changes of the cells of the grey matter (Kress et al. 2014). Once concerning aspect, that has not yet been clarified, is the amount of brain swelling due to the suggested expansion of ECS during sleep.

The extracellular matrix of the brain contains long, unbranched polysaccharide chains, each composed of repeating disaccharide units. One of the disaccharide units is an amino sugar: *N*-acetyl glucosamine or *N*-acetyl galactosamine; collectively, the polysaccharide chains are therefore named glycosaminoglycans (GAGs). GAG chains are negatively charged due to the presence of carboxyl, hydroxyl and sulphate groups. GAGs are hydrophilic due to their highly negatively charged groups; they attract water and positive ions, particularly sodium, contributing to the composition of interstitial fluid.

Aquaporins (AQP) are water-selective channels that regulate osmotically driven water transport through cell membranes. AQP1 is present on the epithelial cells of the choroid plexus, AQP4 is present on the astrocyte foot processes and ependymal cells and AQP9 is localized in tanycytes and astrocyte end-feet. Along with cotransporters of ions and neurotransmitters located on astroglia and neurons, AQP are key factors in regulating water homoeostasis in the ECS. Diffusion is faster in mice lacking aquaporin 4 (AQP4), indicating that the movement of solutes is inversely proportional to the water content of the ECS (Nagelhus and Ottersen 2013). APQ4 is highly expressed in the astrocytic end-feet, and appears to be crucial for the exchanges between the CSF and ISF, as deletion of AQP4 results in a decrease of movement along the walls of arteries into the parenchyma (Iliff et al. 2012). Recent results also show dysregulation of polarization around penetrating arteries in the ageing brain (Kress et al. 2014). In the latter study, the loss of AQP4 polarization during ageing was associated with impairment of the CSF to circulate at these locations after cisternal injections, highlighting the contribution of this channel to CSF circulation in the brain. It should be noted, however, that deletion of AQP4 may alter CSF production by the ependymal cells (Li et al. 2009).

Diffusional properties of the brain are altered by disease. Confocal microscopy studies using fluorescently labelled dextrans show that diffusion in the cortex is reduced in ischaemia (Hrabetova et al. 2003; Sherpa et al. 2014). There is some controversy regarding the role for hydrostatic pressure-driven water flow through astrocyte end-feet, compared to osmotic pressure gradients (Thrane et al. 2015; Smith et al. 2015). As shown by in vivo magnetic resonance imaging studies, APP transgenic mice have a reduced ECS diffusion coefficient compared to age-matched controls. This reduction is more obvious in cortical areas that possess more frequent and severe amyloid deposits along cerebral arteries as CAA, and not significant in the striatum where Aβ deposits are few and diffuse in nature (Mueggler et al. 2004). Transplanting embryonic neural cells from transgenic and wild-type mice into the neocortex and hippocampus of wild-type and APP23 young transgenic hosts, resulted in early amyloid deposition only in the transplants in young transgenic hosts. This suggests that diffusion of soluble Aβ into the ECS of the graft is dependent on the pre-existent pathology in the ECS of the host. Thus, it becomes clear that ageing and neuropathological conditions alter the geometry of the ECS, leading to altered diffusion (Meyer-Luehmann et al. 2003).

The main sources of ISF are the blood and CSF. Water generated as a result of oxidation of glucose to CO_2_ could provide a 10 % contribution to the total volume of ISF, but transport from blood across the endothelium could also be responsible (Abbott 2004). It has been proposed that a large fraction of ISF is derived from the blood, through the capillary endothelium, driven by the Na, K ATPase, with water following passively. In addition to water, ISF consists of tissue metabolites and secreted proteins, including Aβ. Bulk flow of ISF occurs along perivascular pathways and axon tracts. The rate of bulk flow has been reported to be 0.1–0.3 µl min^−1^ g^−1^ in the rat brain (Abbott 2004). It has been suggested that the water crossing the BBB may in fact be taken up by astrocytes, as they express high levels of AQP4. Tracer experiments and mathematical models show that ISF is eliminated from the brain by bulk flow and this occurs along white matter axon tracts and perivascular pathways (Schley et al. 2006; Cserr et al. 1981, 1977).

#### Evidence for Drainage of ISF Along Perivascular Pathways

Tracers such as horseradish peroxidase, Indian ink and I^131^-albumin injected into the brain parenchyma enter perivascular pathways. Earlier studies showed that ISF movement within the perivascular pathways was slow and may have a variable direction compared to the direction of blood flow in the lumen of blood vessels (Rennels et al. 1985). Physiological studies in which a variety of tracers were injected into mammalian brains suggest that size, shape or charge of the tracer is more likely to influence the rate of drainage than molecular weight, confirming that ISF elimination from the brain is by bulk flow.

Tracer experiments were extremely valuable in highlighting the physiological role of perivascular drainage but, due to the absence of confocal and multiphoton microscopy, did not provide clear information regarding the exact position of the ISF tracer in the vessel wall Cserr et al. (1981, 1977). Injections of solutes into the cerebral hemispheres at appropriate concentrations have focused on the kinetics of the removal of tracer from the brain and not on the anatomical route of the elimination pathways. As drainage of proteins and fluid from the brain occurs along blood vessel walls and appears to reach cervical lymph nodes, it is necessary to describe what is known about the structure of blood vessel walls and their perivascular spaces, including their cellular content.

### Structure of Cerebral Blood Vessels

The brain is supplied by the paired internal carotid arteries and vertebral arteries. The arteries lie within the SAS and form the circle of Willis at the base of the brain. Some branches of the circle of Willis supply the deep grey matter structures such as the basal ganglia and thalamus, while branches of the leptomeningeal arteries penetrate the surface of the brain. The latter arteries bifurcate into cortical arteries, which further branch into arterioles and capillaries supplying the cerebral cortex. It is of importance to emphasize that cerebral arteries may differ in structure. The particular structure of these arteries could influence the pathway followed by ISF, waste products and tracers injected experimentally in the brain parenchyma. Excellent reviews that comprise the particular characteristics and changes in arterial structure during neurodegeneration are presented by (Weller et al. 2009a; Weller 2005). Individually, the blood vessels in the mouse have a similar structure to those in the human brain (Lee 1995).

#### Capillaries

Capillary walls in the human brain are formed by an endothelium and a basement membrane, separating it from the brain ECS. The junctions between adjacent endothelial cells form *zonulae occludentes* that are the morphological basis for the BBB. Fibrous astrocytes form end-feet that completely surround the capillary surface: 40–100 nm separate the astrocyte end-feet from the endothelium and this space is occupied by basement membrane (Engelhardt and Coisne 2011). The layer of basement membrane adjacent to the endothelial cell and the layer of basement membrane adjacent to the astrocyte end-feet are fused. The capillary basement membrane is in direct communication with the brain extracellular spaces (Fig. 1). Moreover, the capillary basement membrane is also shared with pericytes, contractile cells that together with the endothelial cells and astrocytes are involved in the regulation of the BBB permeability (Winkler et al. 2011). Further on, the active role of the contractile pericytes in the regulation of capillary diameter and of cerebral blood flow in response to neural activity has been experimentally validated recently (Bell et al. 2010; Peppiatt et al. 2006).

### Arteries

A normal cerebral artery wall has an endothelium separated from layers of smooth muscle cells by basement membrane. Basement membrane is also interposed between individual smooth muscle cells, and between the smooth muscle layers and the leptomeningeal sheath, comparable to the adventitia in systemic arteries. The basement membrane of the glia limitans separates the astrocyte end-feet from the leptomeningeal sheath. The smooth muscle cells within the arterial wall present contractile properties of fundamental importance for the regulation of cerebral blood flow. They are able to respond to mechanical (pressure), electrical, chemical and hormonal stimuli (Lacolley et al. 2012). The adhesion of the vascular cells to their basement membranes is essential for maintaining the integrity of the arterial wall and for recognizing and integrating the high variety of signals that modulate the cell function (Stegemann et al. 2005).

#### Vascular Basement Membranes

Vascular basement membranes are 100 nm laminar matrices produced by endothelial and epithelial cells. The smooth muscle cells from the arterial wall also produce and organize their own basement membrane. In addition to providing a potential pathway for the clearance of solutes out of the brain, the extracellular matrix, including the vascular basement membrane, determines the mechanical properties of the vessel wall and controls the migration and differentiation of vascular cells. In the brain, basement membranes have been reported to be secreted by meningeal cells and contribute to the migration and final positioning of neurons and to the differentiation of the laminar cortical pattern during development (Wagenseil and Mecham 2009; Morris et al. 2014). Basement membranes are composed mainly of collagen type IV, laminin, fibronectin and heparan sulphate proteoglycans. The basement membrane is a dynamic complex, capable of remodelling itself. In vitro, type IV collagen, laminin and fibronectin are capable of assembly into a protein network resembling basement membranes and are interdependent in the formation of the basement membranes (Yurchenco and Schittny 1990).

### Meninges and Subarachnoid Space

The meninges contribute to the preservation of CNS homoeostasis, and constitute of three membranes: the dura mater being the outermost, followed by the arachnoid and the pia mater as the closest to the brain. The SAS is the interval between the arachnoid and the pia mater. The SAS forms cisterns, when the pia mater follows the brain curvatures and separates from the arachnoid. The cisterna pontis and the cisterna magna are the biggest, but other smaller cisterns are found in the SAS. The SAS includes not only CSF, but also the leptomeningeal vessels and the arachnoid trabeculae. The latter extend between the arachnoid and the pia mater. They cushion the brain and protect the cerebral blood vessels from rupture. Moreover, the trabeculae might be considered as an anatomical obstacle in the pathway of the CSF flow. Because of the complex geometry, many modelling studies neglected the presence of the trabeculae. An extensive network of lymphatic vessels, with role in draining solutes from the brain into the deep cervical lymph nodes, has been observed in the mouse brain meninges (Aspelund et al. 2015; Louveau et al. 2015; Andres et al. 1987).

### Perivascular Spaces

The perivascular spaces were described by Virchow and Robin as being in direct communication with the ECS of the brain and with the SAS. This communication between the extracellular brain compartment and the SAS provided a basis for the interpretation that oedema fluid cleared into CSF. The description of the classical Virchow–Robin space has been subsequently challenged and modified. Studies of human brain show that leptomeningeal cells, identified by the presence of desmosomes and small nexus junctions, are reflected from the surface of the brain to coat arteries and veins in the SAS thus separating CSF in the SAS from brain and perivascular spaces (Weller 2005). Furthermore, leptomeningeal cells form a perivascular sheath around blood vessels in the brain. Peri-arterial spaces are separate from the SAS and subpial space; however, venous perivascular spaces communicate with the subpial space. Cerebral peri-arterial spaces are in direct continuation with peri-arterial spaces of leptomeningeal vessels that continue as a layer of perivascular (adventitial) connective tissue coating arteries as they pass through the skull. As the pia mater separates the SAS from the potential perivascular spaces, we do not know whether this has an effect on the composition of the CSF passing through the pia. The pia mater stops particles and, probably, neurotransmitter entering perivascular spaces, but the exact degree of selective permeability of the pia mater and underlying glia limitans is not known.

As mentioned earlier, the structure of the arterial wall and perivascular space differs according to the type of artery. A cortical artery has a narrow perivascular space and does not have an elastic lamina, whereas an artery in the SAS has a large perivascular space and often has an internal elastic lamina. There are also differences between perivascular spaces in the human cerebral cortex and the basal ganglia, as perivascular spaces in the basal ganglia are enclosed by two layers of leptomeninges. The anatomical features of the perivascular spaces in laboratory animals are not well described.

### The Perivascular and Paravascular Clearance Pathways of the Brain

In a very elegant set of experiments, NG2-DS transgenic mice displaying fluorescent vascular smooth muscle cells were injected with tracers in the SAS of the cisterna magna and the pattern of distribution of the tracers was assessed at 30 min after injections were completed (Iliff et al. 2012). The compartment with observable fluorescent soluble tracers was along arterial walls, but the exact anatomical location in relation to the individual components of the arterial wall is still unclear. Although conventional histology was not performed, it appears that the tracers are present in the compartment enclosed between the pia mater and glia limitans, enclosing the vascular wall, termed paravascular. Paravascular entry of soluble tracers from the CSF appears to be dependent on the expression of AQP4 and on efficient arterial pulsations. Once soluble tracers reach the brain parenchyma, they disperse and a proportion is associated with the walls of veins (Iliff et al. 2012, 2013b). Small lipophilic tracers of molecular weights of under 1 kDa selectively behave slightly differently when they move from the CSF into the paravascular compartment, as they appear to follow spatially restricted pathways through the parenchyma. The expression of AQP4 does not affect this process (Rangroo Thrane et al. 2013).

The movement of CSF along the para-arterial compartment into the brain probably represents a slow equilibration system between the CSF and ISF compartments, relying on individual properties of diffusion of pia mater and glia limitans. This equilibration process is not sufficient to explain the changes observed in the CSF in neurodegenerative diseases such as Alzheimer’s disease, where a decrease in the concentration of Aβ in CSF correlates with the severity of disease (Pirttila et al. 1996).

**Fig. 2:**
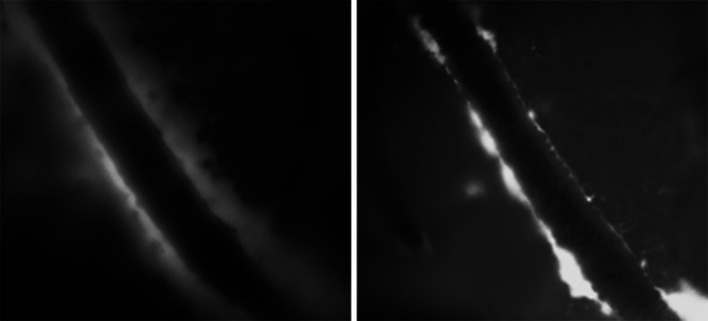
Morphology of the spaces around arteries as observed by fluorescence microscopy. *Left panel*: the dimensions of the space around a cortical artery in vivo in the brain of a mouse. *Right panel*: the same vessel immediately after sacrifice of the mouse, showing collapse of the paravascular space. Scale bar represents 50 μm (Bakker, Levitski and VanBavel, unpublished data)

In contrast to the paravascular pathway outlined by the studies mentioned above (Iliff et al. 2012), data from the Carare–Weller group suggest an alternative route along the vasculature. Thus, in order to define the pathway for the bulk flow drainage of ISF and solutes from the brain parenchyma, minute quantities (0.5 μl) of fluorescent dextran 3 KDa or ovalbumin 49 KDa were injected into the grey matter of the caudate putamen in the centre of the mouse cerebral hemisphere (Carare et al. 2008). By 5 min after injection, tracers had spread diffusely through the ECS but tracer was also present in the walls of blood vessels. Confocal microscopy showed that tracers were co-localized with laminin in the basement membranes of capillaries and in the basement membranes in the tunica media of arteries (Carare et al. 2008). The location of the tracers in artery walls was very specific as tracer was only in the basement membranes between the smooth muscle cells in the tunica media and not in the endothelial basement membranes nor in the outer basement membrane encompassing the artery wall (Hawkes et al. 2014). Some of the tracer, particularly 3KDa dextran, was taken up by a few smooth muscle cells in the tunica media and by perivascular macrophages on the outer aspects of the artery walls. By 30 min after injection, the tracers had disappeared from the ECS of the brain and from the basement membranes in the walls of capillaries and arteries, but tracers remained in the perivascular macrophages (Carare et al. 2008). By 24 h after injection, the route taken by the tracers on their passage out of the brain was outlined by perivascular macrophages containing tracer adjacent to intracerebral arteries and around leptomeningeal arteries on the surface of the brain (Carare et al. 2008). This route of drainage of solutes appears to be fast and specific to solutes, as particles in the range of 20 nm–1 μm do not drain along basement membranes, but rather are engulfed by perivascular macrophages (Zhang et al. 1992; Carare et al. 2008). Peri- and para-vascular spaces along vessels may collapse after sacrifice of the animal, providing an underestimation of the actual size of the compartment (Fig. 2).

Multiphoton microscopy imaging of the living mouse brain during pressure injection of fluorescent tracers into the brain parenchyma has successfully overcome this hurdle (Arbel-Ornath et al. 2013). The tracers aligned very fast (as soon as 2 min after injection) to the arterial basement membrane and were shown to fill the perivascular spaces within the SMC layer. With high temporal resolution and minute volume (<0.5 µl per injection) clearance of the tracers could be tracked at the single vessel level and showed very rapid clearance (within 30 min) in a biexponential fashion that would fit with a bulk flow mechanism. Interestingly, the tracers were only evident in perivascular spaces along arteries, but never along veins. Experimental differences with previous reports are that the tracers used were directly injected to the parenchyma, imaged with very high temporal resolution and quantitatively assessed from the real-time data to determine the kinetics in the living brain. Injecting large (2000 kDa) tracers revealed no clearance of the tracers associated with the perivascular space.

Previous studies, using radio-iodinated serum albumin as a tracer, suggest that once ISF and solutes have left the brain, they drain along the tunica media and the tunica adventitia of the major cerebral arteries, through the base of the skull to deep cervical lymph nodes (Szentistvanyi et al. 1984). The presence of radioactive tracer in the walls of intracranial arteries and the absence of tracer in the walls of the carotid artery in the neck (Szentistvanyi et al. 1984) strongly supports the suggestion that ISF and solutes leave the artery walls to drain to adjacent cervical lymph nodes (Weller et al. 2009b). Quantitative studies of lymphatic drainage for the brain have shown that the speed of drainage is comparable to that from other organs (Szentistvanyi et al. 1984). The quantity and volume of tracers injected to outline the true drainage of ISF from grey matter are too small to be detected in the cervical lymph nodes. However, recent experimental evidence from transgenic mice that overexpress amyloid precursor protein demonstrates that the amount of Aβ in the cervical lymph nodes reflects the degree of production of mutant Aβ in the parenchyma (Pappolla et al. 2014). Furthermore, recent work from (Aspelund et al. 2015) shows that tracers injected into the brain parenchyma drain into the deep cervical lymph nodes via dural lymphatic vessels.

#### Motive Force for Perivascular Drainage

The observation that perivascular transport only occurs in living animals and ceases immediately after cardiac arrest suggests that pulsations in artery walls may generate the motive force for the transport of ISF and solutes out of the brain (Carare et al. 2008). Mathematical models indicate that perivascular transport of ISF and solutes may be driven by the contrary (reflection) waves that follow each pulse wave (Schley et al. 2006). The contrary wave travels in the reverse direction to the major pulse wave and in the reverse direction to the flow of blood. If the contrary wave does act as the motive force for the drainage of ISF and solutes, then the model dictates that some form of valve-like action is required in order to prevent reflux during the passage of the major pulse wave along the vessel wall (Schley et al. 2006). If the route of drainage is within basement membranes (Carare et al. 2008), it is possible that the valve action results from changes in conformation of the basement membrane, together with biochemical interactions. Such a change in conformation of the basement membrane may provide the necessary valve action to ensure that ISF and solutes are driven centrifugally along the artery walls and out of the brain (Schley et al. 2006). As vessels age they become stiff, less elastic and arteriosclerotic, particularly in humans (Weller et al. 2009a); and such stiffening may interfere with perivascular drainage of ISF and solutes in elderly individuals (Weller et al. 2009a). (Iliff et al. 2013b) recently showed that a reduction in pulsatility reduces paravascular flow, whereas an increase in pulsatility with dobutamine increased paravascular flow. Thus, although the work of different groups seems to disagree on several points, there is consensus on the importance of arterial pulsations as a motive force.

Recent experimental evidence suggests that focal stroke as well as amyloid accumulation results in a failure of perivascular clearance of solutes in the affected hemisphere (Arbel-Ornath et al. 2013). Thus, it was shown that, immediately after a stroke, there is a marked impairment of clearance along occluded vessels compared to the non-occluded vessels, suggesting that not only pulsations, but also patent blood flow is necessary for perivascular transport. In agreement with this, it has been shown that cerebral hypoperfusion in a mouse model of Alzheimer’s disease leads to accelerated accumulation of Aβ in the walls of leptomeningeal vessels (Okamoto et al. 2012).

#### Removal of Aβ Along Peri- and Para-vascular Pathways

Although it is not yet possible to perform tracer experiments in humans, Aβ and other amyloids can be considered as natural tracers that define the perivascular pathways for the drainage of ISF and solutes from the human brain (Weller et al. 2008; Revesz et al. 2003). Aβ is deposited as insoluble plaques in the brains of elderly humans and it also accumulates in the walls of cerebral arteries and capillaries as CAA (Lowe et al. 2008). Aβ is cleared along with the ISF and as such eventually aggregates in the perivascular basement membranes. It is plausible that a decrease in ISF clearance in the ageing brain predisposes the perivascular basement membranes to higher concentrations of the peptide where it deposited (Kress et al. 2014; Hawkes et al. 2011). This in turn, increases the stiffness of the vessels and may further delay ISF clearance and thus lead to exacerbation of CAA. Indeed, it has been reported that Aβ-expressing transgenic mice, with extensive amyloid pathology, both parenchymal and vascular, exhibit reduced perivascular clearance (Arbel-Ornath et al. 2013).

Deposition of Aβ is particularly severe in the brain in Alzheimer’s disease and is used as one of the pathological criteria for this disease (Lowe et al. 2008). Produced by secretase cleavage of the transmembrane protein amyloid precursor protein (APP) (Selkoe 2001), Aβ is eliminated from the normal brain by several mechanisms. Such mechanisms include degradation in the brain parenchyma by the enzymes neprilysin and insulin-degrading enzyme (Miners et al. 2008) and by absorption into the blood involving the low density lipoprotein receptor protein-1 pathway (Shibata et al. 2000; Bell et al. 2007) and P-glycoprotein (Cirrito et al. 2005). The other major route for elimination of Aβ is along the perivascular lymphatic drainage pathway (Weller et al. 1998, 2008) that has been defined by tracer studies in experimental animals described above (Carare et al. 2008).

The clearance of Aβ along the paravascular pathway was evaluated by Iliff et al., by injecting ^125^I-labelled Aβ in the brain parenchyma. In their studies, they found that Aqp4-null mice cleared ^125^I-labelled Aβ from the brain about 70 % slower than wild-type animals, suggesting a possible role of Aqp4 in the clearance of the peptide (Iliff et al. 2012). Besides, they also showed that the clearance of ^125^I-labelled Aβ is greater during sleep (Xie et al. 2013), and it is significantly impaired in aged brains (Kress et al. 2014). These particular experiments conducted with ^125^I-labelled Aβ however, do not provide definitive evidence of drainage of the peptide along the glymphatic pathway, as the brains need to be homogenized for quantitation. Nevertheless, these results clearly show that clearance of the brain is impaired with ageing and particularly active during sleep. Experiments with labelled Aβ potentially are better suited to delineate its removal pathways from the brain. The work of (Iliff et al. 2012) shows that 1 h after injection, labelled Aβ is found along capillaries and veins, suggesting outflow along this side of the circulation. An alternative explanation may however be that immune cells take up Aβ selectively along veins and retain fluorescent label, as shown by recent data from (Michaud et al. 2013).

One of the advantages of using CAA to study the course of perivascular drainage is that Aβ is deposited in blood vessel walls as insoluble amyloid, so it can easily be located by immunocytochemistry or by the use of fluorescent stains such as thioflavin (Revesz et al. 2003; Vinters et al. 1996). Insoluble Aβ is deposited in the basement membranes of capillaries and intracerebral arteries (Weller et al. 1998, 2008) in exactly the same distribution as the perivascular pathways outlined by the injection of fluorescent tracers in the mouse brain (Carare et al. 2008). In arteries of the human cerebral cortex, Aβ is deposited in the basement membranes surrounding smooth muscle cells in the tunica media but not in the basement membranes of the arterial endothelium, nor in the basement membranes on the outer aspects of the artery wall (Weller et al. 2008). Such sparing of the endothelial basement membranes in CAA may be significant for the preservation of the endothelium itself and avoidance of widespread thrombotic occlusion of arteries with CAA.

Deposits of Aβ in the walls of the medium-sized leptomeningeal arteries are seen in tunica media and in the adventitia (Weller et al. 2009a; Revesz et al. 2003). One intriguing question is: does Aβ drain from brain to cervical lymph nodes in humans? Evidence from biochemical studies of soluble Aβ in the walls of cerebral arteries suggest that it does. Aβ is present in the walls of intracranial arteries, even in young adults, but is not detected in the walls of the carotid artery in the neck (Shinkai et al. 1995). As in the animal studies (Szentistvanyi et al. 1984), this suggests that solutes such as Aβ draining from the brain leave the wall of the carotid artery for the lymph nodes associated with the carotid artery at the base of the skull.

#### Significance of Perivascular Drainage for the Pathophysiology of Hydrocephalus and Syringomyelia

There is an interface between the CSF and ISF not only at the cortical surfaces of the brain but also at the ventricular surfaces. The outer surface of the spinal cord is associated with CSF in the SAS and CSF has access to the central canal particularly in foetal life.

Obstruction to the flow or absorption of CSF may result in hydrocephalus with dilatation of the cerebral ventricular system. In the acute stages of hydrocephalus, particularly in young children and young animals, periventricular oedema occurs due to transependymal infusion of CSF from the ventricles into the white matter. Grey matter areas adjacent to the ventricles do not show such oedema (Weller 1998). Lesser degrees of periventricular oedema occur in adults with hydrocephalus and are detectable by MRI; ventricular shunting or treatment with acetazolamide that reduces the production of CSF both decrease the severity of the periventricular oedema in acute hydrocephalus (Alperin et al. 2014). It appears that perivascular drainage of fluid from the white matter is not able to adequately compensate for blockage of CSF drainage. So, not only do the ventricles dilate with accumulated CSF but fluid infused into periventricular white matter does not drain adequately out of the brain by the perivascular route.

Similar accumulation of CSF is seen in syringomyelia. Fluid collects in a syrinx within the spinal cord with oedema of surrounding cord tissue (Harding and Copp 2008); drainage mechanisms within the spinal cord parenchyma do not appear to be adequate to drain such fluid. One factor responsible for the formation of the syrinx in the cervical spinal cord appears to be disturbance of normal free flow of CSF through the foramen magnum with consequent flow of CSF down the central canal of the spinal cord (Wetjen et al. 2008). It has also been proposed that CSF enters the spinal cord from an obstructed SAS alongside arteries, spreads through the parenchyma and accumulates in the syrinx in the centre of the spinal cord (Bilston et al. 2010).

Drainage of fluid from cerebral white matter can also be compromised by blocking peri-arterial drainage pathways with CAA. Dilatation of perivascular spaces in the white matter can be observed by MRI in the elderly population and in Alzheimer’s disease (Charidimou et al. 2015). Imaging and pathological studies suggest that dilated perivascular spaces correlate with the severity of CAA in cortical and leptomeningeal arteries (Charidimou et al. 2015; Roher et al. 2003).

#### Controversy Regarding the Direction of Peri- and Para-vascular Flow

**Fig. 3:**
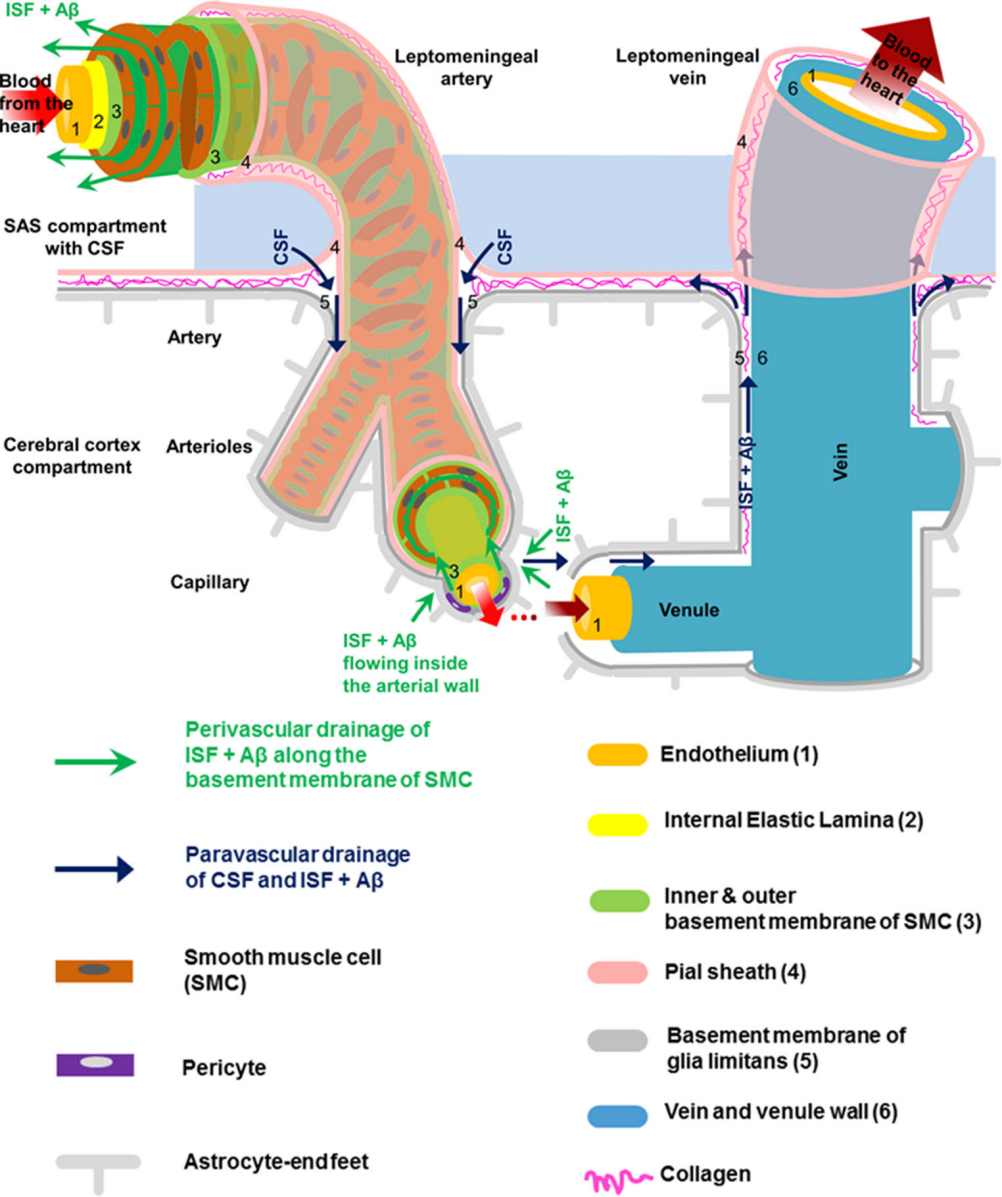
Schematic representation of the peri- and para-vascular spaces in the human cerebral cortex (not to scale). The subarachnoid space (SAS), filled with cerebrospinal fluid (CSF), is separated from the subpial space by the pia matter (Alcolado et al. 1988). A leptomeningeal sheath (the pial sheath shown in *light pink*) derived from the pia matter is also reflected on the walls of both arteries and veins that are crossing the SAS, thus separating the SAS from the cerebral cortex. Bundles of collagen are interposed in the subpial space and form the adventitia of leptomeningeal blood vessels, which is an expandable perivascular space. The pial sheath surrounding the leptomeningeal arteries in SAS continues to coat the arteries as they penetrate the cortex and it is closely applied to the outer basement membrane of smooth muscle cells (SMCs). Nonetheless, the veins from the cerebral cortex do not have a coating pial sheath. The middle layers of basement membrane (*dark green*), situated between layers of SMCs, represent the pathway for the perivascular drainage of solutes out of the brain. As emphasized, solutes do not drain along the inner or outer basement membrane layers of SMCs (*light green*). For the sake of simplicity, we illustrated only one layer of SMCs spiralling within the arterial wall. The perivascular drainage pathway (*green arrows*) follows a similar spiral trajectory which is spanned by a matrix of proteins. The basement membrane of the glia limitans (*dark grey*) is closely applied to the pia matter as the artery enters the brain. Thus another narrow perivascular space is created between the irregular surface of the pial sheath and the outer basement membrane of the SMCs and that of the glia limitans, respectively (Zhang et al. 1990). The narrow perivascular space has also been presented under the nomenclature of “paravascular space” and it has been proposed to be part of the glymphatic pathway (*dark blue arrows*). The cortical para-arterial pathway was referred to as the Virchow–Robin space (VRS). However, in the era of electron microscopy the exact boundaries of the VRS are not clearly defined (Color figure online)

A salient issue in the field of peri- and para-vascular flow is the apparent discrepancy regarding the direction of these two types of flow. Early papers described a back and forth longitudinal movement of dye, which was slow and variable in direction (Ichimura et al. 1991). On the other hand, other work suggested that CSF flows from the SAS in the perivascular space of penetrating arteries, from where it reaches the basal lamina of capillaries and then flows into the brain parenchyma. From there, it returned to the basal lamina of venules or in the perivascular space of large veins (Rennels et al. 1985). Thus, a paravascular inflow of CSF into the brain along arteries, which mixes with ISF and leaves the brain along veins, has been proposed (Iliff et al. 2012). Alternatively, a perivascular route using the extracellular spaces along basement membranes within the walls of capillaries and arteries that exits the brain, in the direction opposite to the arterial blood flow has been suggested (Carare et al. 2008) (Fig. 3).

One possible explanation for these opposite views is that two pathways exist, being separated by perivascular meningeal sheets. It would however be rather unlikely that two pathways exist for drainage of the brain, flowing in opposite directions. An alternative explanation however, is that the same pathway is described, but flow directions are differently interpreted because of methodological differences. It can be envisioned that the pulsations along arteries create a mixing action in the peri-/para-vascular compartment, but do not provide a directional force for flow per se. Tracers, entering the compartment from the parenchyma may then spread out of the brain, creating the impression of flow retrograde to arterial inflow. On the other hand, tracers injected in the CSF (usually the cisterna magna) may spread into the brain along the same peri-/para-vascular spaces, again as a result of mixing by arterial and CSF pulsations. This could be interpreted as inflow into the brain along arteries, following the same direction of blood flow. Indeed, in most studies in which parenchymal injections of tracers were used, it was concluded that perivascular flow is outward (Carare et al. 2008), while in the studies in which tracers were injected into the cisterna magna, inward flow was observed (Iliff et al. 2012). There are exceptions to this notion, as it was found that labelled Aβ, was detected along capillaries and veins after parenchymal injection (Iliff et al. 2012). But in that case, the initial route of elimination out of the brain was not determined. Thus, it is possible that differences in approach may at least partially explain the different findings regarding the direction of the peri-/para-vascular flow. The idea of mixing within the CSF compartment is actually more in agreement with earlier work, where it was found that flow in the perivascular space is pulsatile and variable in direction (Ichimura et al. 1991).

In all cases, the injection of tracers into the brain is very sensitive to pressure and volume disturbances. In the paper where the brain’s drainage system was coined as the glymphatic pathway, tracers were injected at a rate of 2 μl/min, injecting a total volume of 10 μl (Iliff et al. 2012). In mice, total CSF production is estimated as <1 μl/min (Rudick et al. 1982), with a total volume of CSF around 37 μl. Thus, the normal direction of flow in this compartment may easily be overwhelmed in such cases, as it was also pointed out in recent review (Hladky and Barrand 2014). In addition to the concerns raised in that paper, other points can be made regarding the concept of glymphatic drainage.

Firstly, the removal of Aβ along veins does not correlate with Aβ preferentially depositing along arteries, as is particularly noticeable in CAA.

Secondly, how does the pulsation of arteries provide directionality to the paravascular flow? Pulsations alone of the paravascular space are not expected to create flow. A mixing action could however be anticipated, as described above. There appears to be an analogy with the circulation of blood, but in this case there is no entity identified yet that fulfils the role of the ‘heart’ as a pump.

Thirdly, when studying the impact of sleep and anaesthesia on paravascular flow, it was postulated that variations in the parenchymal extracellular volume during sleep, anaesthesia or infusion of a norepinephrine antagonist determine paravascular inflow. However, could some of the conditions studied not only alter the dimensions of interstitial spaces, but also CSF production in general? Thus, at least in humans, sleep is known to be associated with increased CSF production (Nilsson et al. 1992). In this manner, paravascular flow and CSF–ISF exchange may not only change because of effects on interstitial conductance, but also because of a change in CSF production, which provides an input into the system.

Fourth, tracers with different molecular size were used in several studies. Not unexpectedly, large tracers were found not to penetrate the brain from the paravascular space. However, when followed in time, large tracers would then be expected to show a different rate of clearance from the brain, or even accumulate in the paravascular space due to a sieving action. This was not observed, as the kinetics of removal from the brain was similar for the small and large tracers (Iliff et al. 2013a).

Lastly, parts of the anatomical basis for the glymphatic pathway remain unclear. When injected in the CSF, tracers follow a “physically and functionally distinct subcompartment” of the SAS (Iliff et al. 2012), but the complete and exact route of drainage of interstitial fluid and its dynamics in relation to time is still unclear.

## Conclusions

In this paper, we have reviewed the pathways for lymphatic drainage of CSF that are largely separated from the perivascular pathways for lymphatic drainage of ISF from the brain parenchyma. Interrelationships between CSF and ISF have also been emphasized. Combining physiological studies with examination of the anatomical pathways for lymphatic drainage of the brain and CSF is essential for understanding lymphatic clearance in health and disease. The recent discovery of the dural lymphatic vessels in the mouse brain (Aspelund et al. 2015; Louveau et al. 2015) brings new insights on how ISF/CSF from SAS drains into the cervical lymph nodes. However, the puzzle of the entire lymphatic system of the brain is still far from being completely solved. Some missing pieces concern the lymphatic system within the parenchyma and the existence of an equivalent dural lymphatic system in the human brain. The importance of understanding the clearance of solutes from the brain goes beyond neurodegenerative diseases. Considering that the BBB is not easily penetrated by the therapeutic drugs for neurological disorders, additional methods for delivering the drugs into the brain parenchyma, such as direct drug administration to the CSF or to the brain parenchyma have been employed (Barua et al. 2012; Sampson et al. 2011). However, infusion of therapeutic drugs in certain areas of the brain could result in clearance of the drugs along the proposed peri/paravascular clearance pathways described above, instead of being delivered to the targeted tissue (Brady et al. 2013). Therefore, elucidating the clearance pathways of solutes from the brain is of paramount importance not only for understanding the development of neurodegenerative disease, but also for successful delivery of therapeutic drugs to the brain tissue targeted for therapy.

## References

[CR1] Abbott NJ (2004) Evidence for bulk flow of brain interstitial fluid: significance for physiology and pathology. Neurochem Int 45(4):545–552 15186921 10.1016/j.neuint.2003.11.006

[CR2] Abbott NJ (2013) Blood–brain barrier structure and function and the challenges for CNS drug delivery. J Inherit Metab Dis 36(3):437–449 23609350 10.1007/s10545-013-9608-0

[CR3] Alcolado R, Weller R, Parrish E, Garrod D (1988) The cranial arachnoid and pia mater in man: anatomical and ultrastructural observations. Neuropathol Appl Neurobiol 14(1):1–17 3374751 10.1111/j.1365-2990.1988.tb00862.x

[CR4] Alperin N, Oliu CJ, Bagci AM, Lee SH, Kovanlikaya I, Adams D, Katzen H, Ivkovic M, Heier L, Relkin N (2014) Low-dose acetazolamide reverses periventricular white matter hyperintensities in iNPH. Neurology 82(15):1347–1351 24634454 10.1212/WNL.0000000000000313 PMC4001191

[CR5] Andres K, Von Düring M, Muszynski K, Schmidt R (1987) Nerve fibres and their terminals of the dura mater encephali of the rat. Anat Embryol 175(3):289–301 3826655 10.1007/BF00309843

[CR6] Arbel-Ornath M, Hudry E, Eikermann-Haerter K, Hou S, Gregory JL, Zhao L, Betensky RA, Frosch MP, Greenberg SM, Bacskai BJ (2013) Interstitial fluid drainage is impaired in ischemic stroke and Alzheimer’s disease mouse models. Acta Neuropathol 126(3):353–364. doi:10.1007/s00401-013-1145-2 23818064 10.1007/s00401-013-1145-2 PMC3810119

[CR7] Aspelund A, Antila S, Proulx ST, Karlsen TV, Karaman S, Detmar M, Wiig H, Alitalo K (2015) A dural lymphatic vascular system that drains brain interstitial fluid and macromolecules. J Exp Med. doi:10.1084/jem.20142290 26077718 10.1084/jem.20142290 PMC4493418

[CR8] Barua NU, Miners JS, Bienemann AS, Wyatt MJ, Welser K, Tabor AB, Hailes HC, Love S, Gill SS (2012) Convection-enhanced delivery of neprilysin: a novel amyloid-β-degrading therapeutic strategy. J Alzheimers Dis 32(1):43–56 22751177 10.3233/JAD-2012-120658

[CR9] Bell RD, Sagare AP, Friedman AE, Bedi GS, Holtzman DM, Deane R, Zlokovic BV (2007) Transport pathways for clearance of human Alzheimer’s amyloid beta-peptide and apolipoproteins E and J in the mouse central nervous system. J Cereb Blood Flow Metab 27:909–918 17077814 10.1038/sj.jcbfm.9600419 PMC2853021

[CR10] Bell RD, Winkler EA, Sagare AP, Singh I, LaRue B, Deane R, Zlokovic BV (2010) Pericytes control key neurovascular functions and neuronal phenotype in the adult brain and during brain aging. Neuron 68(3):409 21040844 10.1016/j.neuron.2010.09.043 PMC3056408

[CR11] Bilston LE, Stoodley MA, Fletcher DF (2010) The influence of the relative timing of arterial and subarachnoid space pulse waves on spinal perivascular cerebrospinal fluid flow as a possible factor in syrinx development: laboratory investigation. J Neurosurg 112(4):808–813 19522574 10.3171/2009.5.JNS08945

[CR12] Bozanovic-Sosic R, Mollanji R, Johnston MG (2001) Spinal and cranial contributions to total cerebrospinal fluid transport. Am J Physiol Regul Integr Comp Physiol 281:R909–R916 11507008 10.1152/ajpregu.2001.281.3.R909

[CR13] Brady ML, Raghavan R, Alexander A, Kubota K, Sillay K, Emborg ME (2013) Pathways of infusate loss during convection enhanced delivery into the putamen nucleus. Stereotact Funct Neurosurg 91(2):69 23344643 10.1159/000342492 PMC3716010

[CR14] Brinker T, Stopa E, Morrison J, Klinge P (2014) A new look at cerebrospinal fluid circulation. Fluids Barriers CNS 11:10. doi:10.1186/2045-8118-11-10 24817998 10.1186/2045-8118-11-10 PMC4016637

[CR15] Carare R, Bernardes-Silva M, Newman T, Page A, Nicoll J, Perry V, Weller R (2008) Solutes, but not cells, drain from the brain parenchyma along basement membranes of capillaries and arteries: significance for cerebral amyloid angiopathy and neuroimmunology. Neuropathol Appl Neurobiol 34(2):131–144 18208483 10.1111/j.1365-2990.2007.00926.x

[CR16] Charidimou A, Hong YT, Jäger HR, Fox Z, Aigbirhio FI, Fryer TD, Menon DK, Warburton EA, Werring DJ, Baron J-C (2015) white matter perivascular spaces on magnetic resonance imaging marker of cerebrovascular amyloid burden? Stroke 46(6):1707–1709 25908461 10.1161/STROKEAHA.115.009090

[CR17] Cirrito JR, Deane R, Fagan AM, Spinner ML, Parsadanian M, Finn MB, Jiang H, Prior JL, Sagare A, Bales KR, Paul SM, Zlokovic BV, Piwnica-Worms D, Holtzman DM (2005) P-glycoprotein deficiency at the blood-brain barrier increases amyloid-beta deposition in an Alzheimer disease mouse model. J Clin Invest 115:3285–3290 16239972 10.1172/JCI25247 PMC1257538

[CR18] Cserr HF, Cooper D, Milhorat T (1977) Flow of cerebral interstitial fluid as indicated by the removal of extracellular markers from rat caudate nucleus. Exp Eye Res 25:461–473 590401 10.1016/s0014-4835(77)80041-9

[CR19] Cserr H, Cooper D, Suri P, Patlak C (1981) Efflux of radiolabeled polyethylene glycols and albumin from rat brain. Am J Physiol Ren Physiol 240(4):F319–F328 10.1152/ajprenal.1981.240.4.F319 7223889

[CR20] Damkier HH, Brown PD, Praetorius J (2013) Cerebrospinal fluid secretion by the choroid plexus. Physiol Rev 93(4):1847–1892. doi:10.1152/physrev.00004.2013 24137023 10.1152/physrev.00004.2013

[CR21] de Lange EC (2004) Potential role of ABC transporters as a detoxification system at the blood-CSF barrier. Adv Drug Deliv Rev 56(12):1793–1809. doi:10.1016/j.addr.2004.07.009 15381334 10.1016/j.addr.2004.07.009

[CR22] Di Chiro G (1964) Movement of the cerebrospinal fluid in human beings. Nature (Lond) 204:290–291 14212432 10.1038/204290a0

[CR23] Engelhardt B, Coisne C (2011) Fluids and barriers of the CNS establish immune privilege by confining immune surveillance to a two-walled castle moat surrounding the CNS castle. Fluids Barriers CNS 8(4):10–1186 21349152 10.1186/2045-8118-8-4 PMC3039833

[CR24] Enzmann DR, Pelc N (1993) Cerebrospinal fluid flow measured by phase-contrast cine MR. Am J Neuroradiol 14(6):1301–1307 8279323 PMC8367526

[CR25] Harding B, Copp A (2008) Malformations

[CR26] Hawkes CA, Härtig W, Kacza J, Schliebs R, Weller RO, Nicoll JA, Carare RO (2011) Perivascular drainage of solutes is impaired in the ageing mouse brain and in the presence of cerebral amyloid angiopathy. Acta Neuropathol 121(4):431–443 21259015 10.1007/s00401-011-0801-7

[CR27] Hawkes CA, Jayakody N, Johnston DA, Bechmann I, Carare RO (2014) Failure of perivascular drainage of beta-amyloid in cerebral amyloid angiopathy. Brain Pathol 24(4):396–403. doi:10.1111/bpa.12159 24946077 10.1111/bpa.12159 PMC8029317

[CR28] Hladky SB, Barrand MA (2014) Mechanisms of fluid movement into, through and out of the brain: evaluation of the evidence. Fluids Barriers CNS 11(1):1–32 25678956 10.1186/2045-8118-11-26 PMC4326185

[CR29] Hrabětová S, Nicholson C (2004) Contribution of dead-space microdomains to tortuosity of brain extracellular space. Neurochem Int 45(4):467–477 15186912 10.1016/j.neuint.2003.11.011

[CR30] Hrabetova S, Hrabe J, Nicholson C (2003) Dead-space microdomains hinder extracellular diffusion in rat neocortex during ischemia. J Neurosci 23(23):8351–8359 12967997 10.1523/JNEUROSCI.23-23-08351.2003 PMC6740703

[CR31] Ichimura T, Fraser PA, Cserr HF (1991) Distribution of extracellular tracers in perivascular spaces of the rat brain. Brain Res 545(1–2):103–113 1713524 10.1016/0006-8993(91)91275-6

[CR32] Iliff JJ, Wang M, Liao Y, Plogg BA, Peng W, Gundersen GA, Benveniste H, Vates GE, Deane R, Goldman SA, Nagelhus EA, Nedergaard M (2012) A paravascular pathway facilitates CSF flow through the brain parenchyma and the clearance of interstitial solutes, including amyloid beta. Sci Transl Med 4(147):147ra111. doi:10.1126/scitranslmed.3003748 22896675 10.1126/scitranslmed.3003748 PMC3551275

[CR33] Iliff JJ, Lee H, Yu M, Feng T, Logan J, Nedergaard M, Benveniste H (2013a) Brain-wide pathway for waste clearance captured by contrast-enhanced MRI. J Clin Investig 123(3):1299–1309. doi:10.1172/JCI67677 23434588 10.1172/JCI67677 PMC3582150

[CR34] Iliff JJ, Wang M, Zeppenfeld DM, Venkataraman A, Plog BA, Liao Y, Deane R, Nedergaard M (2013b) Cerebral arterial pulsation drives paravascular CSF-interstitial fluid exchange in the murine brain. J Neurosci 33(46):18190–18199. doi:10.1523/JNEUROSCI.1592-13.2013 24227727 10.1523/JNEUROSCI.1592-13.2013 PMC3866416

[CR35] Johnston M, Zakharov A, Papaiconomou C, Salmasi G, Armstrong D (2004) Evidence of connections between cerebrospinal fluid and nasal lymphatic vessels in humans, non-human primates and other mammalian species. Cerebrospinal fluid research 1(1):2. doi:10.1186/1743-8454-1-2 15679948 10.1186/1743-8454-1-2 PMC546409

[CR36] Kida S, Pantazis A, Weller R (1993) CSF drains directly from the subarachnoid space into nasal lymphatics in the rat. Anatomy, histology and immunological significance. Neuropathol Appl Neurobiol 19(6):480–488 7510047 10.1111/j.1365-2990.1993.tb00476.x

[CR37] Kress BT, Iliff JJ, Xia M, Wang M, Wei HS, Zeppenfeld D, Xie L, Kang H, Xu Q, Liew JA (2014) Impairment of paravascular clearance pathways in the aging brain. Ann Neurol 76(6):845–861 25204284 10.1002/ana.24271 PMC4245362

[CR38] Lacolley P, Regnault V, Nicoletti A, Li Z, Michel J-B (2012) The vascular smooth muscle cell in arterial pathology: a cell that can take on multiple roles. Cardiovasc Res. doi:10.1093/cvr/cvs135 10.1093/cvr/cvs135 22467316

[CR39] Lee RM (1995) Morphology of cerebral arteries. Pharmacol Ther 66(1):149–173 7630927 10.1016/0163-7258(94)00071-a

[CR40] Li X, Kong H, Wu W, Xiao M, Sun X, Hu G (2009) Aquaporin-4 maintains ependymal integrity in adult mice. Neuroscience 162(1):67–77. doi:10.1016/j.neuroscience.2009.04.044 19393298 10.1016/j.neuroscience.2009.04.044

[CR41] Louveau A, Smirnov I, Keyes TJ, Eccles JD, Rouhani SJ, Peske JD, Derecki NC, Castle D, Mandell JW, Lee KS (2015) Structural and functional features of central nervous system lymphatic vessels. Nature. doi:10.1038/nature14432 26030524 10.1038/nature14432 PMC4506234

[CR42] Lowe J, Mirra SS, Hyman BT, Dickson DW (2008) Ageing and dementia. In: Love S, Louis DN, Ellison DW (eds) Greenfield’s neuropathology, vol 1, 8th edn. Hodder Arnold, London, pp 1031–1152

[CR43] Meyer-Luehmann M, Stalder M, Herzig MC, Kaeser SA, Kohler E, Pfeifer M, Boncristiano S, Mathews PM, Mercken M, Abramowski D (2003) Extracellular amyloid formation and associated pathology in neural grafts. Nat Neurosci 6(4):370–377 12598899 10.1038/nn1022

[CR44] Michaud JP, Bellavance MA, Prefontaine P, Rivest S (2013) Real-time in vivo imaging reveals the ability of monocytes to clear vascular amyloid beta. Cell Rep 5(3):646–653. doi:10.1016/j.celrep.2013.10.010 24210819 10.1016/j.celrep.2013.10.010

[CR45] Milhorat TH (1975) The third circulation revisited. J Neurosurg 42:628–642 167134 10.3171/jns.1975.42.6.0628

[CR46] Miners JS, Baig S, Palmer J, Palmer LE, Kehoe PG, Love S (2008) Abeta-degrading enzymes in Alzheimer’s disease. Brain Pathol 18:240–252 18363935 10.1111/j.1750-3639.2008.00132.x PMC8095507

[CR47] Morris AW, Carare RO, Schreiber S, Hawkes CA (2014) The cerebrovascular basement membrane: role in the clearance of β-amyloid and cerebral amyloid angiopathy. Frontiers in aging neuroscience 6 10.3389/fnagi.2014.00251 PMC4168721 25285078

[CR48] Mueggler T, Meyer-Luehmann M, Rausch M, Staufenbiel M, Jucker M, Rudin M (2004) Restricted diffusion in the brain of transgenic mice with cerebral amyloidosis. Eur J Neurosci 20(3):811–817 15255991 10.1111/j.1460-9568.2004.03534.x

[CR49] Nagelhus EA, Ottersen OP (2013) Physiological roles of aquaporin-4 in brain. Physiol Rev 93(4):1543–1562 24137016 10.1152/physrev.00011.2013 PMC3858210

[CR50] Nagra G, Koh L, Zakharov A, Armstrong D, Johnston M (2006) Quantification of cerebrospinal fluid transport across the cribriform plate into lymphatics in rats. Am J Physiol Regul Integr Comp Physiol 291(5):R1383–R1389. doi:10.1152/ajpregu.00235.2006 16793937 10.1152/ajpregu.00235.2006

[CR51] Nicholson C (1992) Quantitative analysis of extracellular space using the method of TMA+ iontophoresis and the issue of TMA + uptake. Can J Physiol Pharmacol 70(Suppl):S314–S322 1295682 10.1139/y92-278

[CR52] Nicholson C, Kamali-Zare P, Tao L (2011) Brain extracellular space as a diffusion barrier. Comput Vis Sci 14(7):309–325 23172993 10.1007/s00791-012-0185-9 PMC3500962

[CR53] Nilsson C, Stahlberg F, Thomsen C, Henriksen O, Herning M, Owman C (1992) Circadian variation in human cerebrospinal fluid production measured by magnetic resonance imaging. Am J Physiol 262(1 Pt 2):R20–R24 1733335 10.1152/ajpregu.1992.262.1.R20

[CR54] Okamoto Y, Yamamoto T, Kalaria RN, Senzaki H, Maki T, Hase Y, Kitamura A, Washida K, Yamada M, Ito H, Tomimoto H, Takahashi R, Ihara M (2012) Cerebral hypoperfusion accelerates cerebral amyloid angiopathy and promotes cortical microinfarcts. Acta Neuropathol 123(3):381–394. doi:10.1007/s00401-011-0925-9 22170742 10.1007/s00401-011-0925-9 PMC3282897

[CR55] Pappolla M, Sambamurti K, Vidal R, Pacheco-Quinto J, Poeggeler B, Matsubara E (2014) Evidence for lymphatic Abeta clearance in Alzheimer’s transgenic mice. Neurobiol Dis 71:215–219. doi:10.1016/j.nbd.2014.07.012 25102344 10.1016/j.nbd.2014.07.012 PMC5589075

[CR56] Peppiatt CM, Howarth C, Mobbs P, Attwell D (2006) Bidirectional control of CNS capillary diameter by pericytes. Nature 443(7112):700–704 17036005 10.1038/nature05193 PMC1761848

[CR57] Pirttila T, McHta PD, Soininen H, Kim KS, Heinonen O, Paljarvi L, Kosunen O, Riekkinen P, Wisniewski HM (1996) Cerebrospinal fluid concentrations of soluble amyloid á-protein and apolipoprotein E in patients with Alzheimer’s disease. Correlations with amyloid load in the brain. Arch Neurol 53:189–193 8639071 10.1001/archneur.1996.00550020105022

[CR58] Rangroo Thrane V, Thrane AS, Plog BA, Thiyagarajan M, Iliff JJ, Deane R, Nagelhus EA, Nedergaard M (2013) Paravascular microcirculation facilitates rapid lipid transport and astrocyte signaling in the brain. Sci Rep 3:2582. doi:10.1038/srep02582 24002448 10.1038/srep02582 PMC3761080

[CR59] Rennels ML, Gregory TF, Blaumanis OR, Fujimoto K, Grady PA (1985) Evidence for a ‘paravascular’ fluid circulation in the mammalian central nervous system, provided by the rapid distribution of tracer protein throughout the brain from the subarachnoid space. Brain Res 326(1):47–63 3971148 10.1016/0006-8993(85)91383-6

[CR60] Revesz T, Ghiso J, Lashley T, Plant G, Rostagno A, Frangione B, Holton JL (2003) Cerebral amyloid angiopathies: a pathologic, biochemical, and genetic view. J Neuropathol Exp Neurol 62(9):885–898 14533778 10.1093/jnen/62.9.885

[CR61] Roher AE, Kuo Y-M, Esh C, Knebel C, Weiss N, Kalback W, Luehrs DC, Childress JL, Beach TG, Weller RO (2003) Cortical and leptomeningeal cerebrovascular amyloid and white matter pathology in Alzheimer’s disease. Mol Med 9(3–4):112 12865947 PMC1430731

[CR62] Rudick RA, Zirretta DK, Herndon RM (1982) Clearance of albumin from mouse subarachnoid space: a measure of CSF bulk flow. J Neurosci Methods 6(3):253–259 7144238 10.1016/0165-0270(82)90088-7

[CR63] Sampson JH, Brady M, Raghavan R, Mehta AI, Friedman AH, Reardon DA, Petry NA, Barboriak DP, Wong TZ, Zalutsky MR (2011) Colocalization of gadolinium-diethylene triamine pentaacetic acid with high-molecular-weight molecules after intracerebral convection-enhanced delivery in humans. Neurosurgery 69(3):668–676 21430586 10.1227/NEU.0b013e3182181ba8 PMC3154565

[CR64] Schley D, Carare-Nnadi R, Please C, Perry V, Weller R (2006) Mechanisms to explain the reverse perivascular transport of solutes out of the brain. J Theor Biol 238(4):962–974 16112683 10.1016/j.jtbi.2005.07.005

[CR65] Selkoe DJ (2001) Alzheimer’s disease: genes, proteins, and therapy. Physiol Rev 81(2):741–766 11274343 10.1152/physrev.2001.81.2.741

[CR66] Sherpa AD, van de Nes P, Xiao F, Weedon J, Hrabetova S (2014) Gliotoxin-induced swelling of astrocytes hinders diffusion in brain extracellular space via formation of dead-space microdomains. Glia 62(7):1053–1065. doi:10.1002/glia.22661 24687699 10.1002/glia.22661 PMC4059363

[CR67] Shibata M, Yamada S, Kumar SR, Calero M, Bading J, Frangione B, Holtzman DM, Miller CA, Strickland DK, Ghiso J, Zlokovic BV (2000) Clearance of Alzheimer’s amyloid-beta(1-40) peptide from brain by LDL receptor-related protein-1 at the blood-brain barrier. J Clin Invest 106(12):1489–1499 11120756 10.1172/JCI10498 PMC387254

[CR68] Shinkai Y, Yoshimura M, Ito Y, Odaka A, Suzuki N, Yanagisawa K, Ihara Y (1995) Amyloid beta -proteins 1-40 and 1-42(43) in the soluble fraction of extra- and intracranial blood vessels. Ann Neurol 38(3):421–428 7668828 10.1002/ana.410380312

[CR69] Smith AJ, Jin BJ, Verkman AS (2015) Muddying the water in brain edema? Trends Neurosci. doi:10.1016/j.tins.2015.04.006 25980601 10.1016/j.tins.2015.04.006 PMC4739497

[CR70] Stegemann JP, Hong H, Nerem RM (2005) Mechanical, biochemical, and extracellular matrix effects on vascular smooth muscle cell phenotype. J Appl Physiol 98(6):2321–2327 15894540 10.1152/japplphysiol.01114.2004

[CR71] Syková E, Nicholson C (2008) Diffusion in brain extracellular space. Physiol Rev 88(4):1277–1340 18923183 10.1152/physrev.00027.2007 PMC2785730

[CR72] Szentistvanyi I, Patlak CS, Ellis RA, Cserr HF (1984) Drainage of interstitial fluid from different regions of rat brain. Am J Physiol 246:F835–F844 6742132 10.1152/ajprenal.1984.246.6.F835

[CR73] Thorne RG, Hrabětová S, Nicholson C (2004a) Diffusion of epidermal growth factor in rat brain extracellular space measured by integrative optical imaging. J Neurophysiol 92(6):3471–3481 15269225 10.1152/jn.00352.2004

[CR74] Thorne RG, Pronk GJ, Padmanabhan V, Frey WH (2004b) Delivery of insulin-like growth factor-1 to the rat brain and spinal cord along olfactory and trigeminal pathways following intranasal administration. Neuroscience 127:481–496 15262337 10.1016/j.neuroscience.2004.05.029

[CR75] Thrane AS, Rangroo Thrane V, Plog BA, Nedergaard M (2015) Filtering the muddied waters of brain edema. Trends Neurosci. doi:10.1016/j.tins.2015.04.009 26008121 10.1016/j.tins.2015.04.009

[CR76] Veening JG, Barendregt HP (2010) The regulation of brain states by neuroactive substances distributed via the cerebrospinal fluid; a review. Cerebrospinal Fluid Res 7:1. doi:10.1186/1743-8454-7-1 20157443 10.1186/1743-8454-7-1 PMC2821375

[CR77] Vinters HV, Wang ZZ, Secor DL (1996) Brain parenchymal and microvascular amyloid in Alzheimer’s disease. Brain Pathol 6(2):179–195 8737932 10.1111/j.1750-3639.1996.tb00799.x

[CR78] Wagenseil JE, Mecham RP (2009) Vascular extracellular matrix and arterial mechanics. Physiol Rev 89(3):957–989 19584318 10.1152/physrev.00041.2008 PMC2775470

[CR79] Weed LH (1923) The absorbtion of cerebrospinal fluid into the venous system. Ame J Anat 31(3):191–221

[CR80] Weller RO (1998) Pathology of cerebrospinal fluid and interstitial fluid of the CNS: significance for Alzheimer disease, prion disorders and multiple sclerosis. J Neuropathol Exp Neurol 57(10):885–894 9786239 10.1097/00005072-199810000-00001

[CR81] Weller R (2005) Microscopic morphology and histology of the human meninges. Morphologie 89(284):22–34 15943078 10.1016/s1286-0115(05)83235-7

[CR82] Weller RO, Massey A, Newman TA, Hutchings M, Kuo YM, Roher AE (1998) Cerebral amyloid angiopathy: amyloid beta accumulates in putative interstitial fluid drainage pathways in Alzheimer’s disease. Am J Pathol 153(3):725–733 9736023 10.1016/s0002-9440(10)65616-7 PMC1853019

[CR83] Weller RO, Subash M, Preston SD, Mazanti I, Carare RO (2008) perivascular drainage of amyloid-beta peptides from the brain and its failure in cerebral amyloid angiopathy and Alzheimer’s disease. Brain Pathol 18:253–266 18363936 10.1111/j.1750-3639.2008.00133.x PMC8095597

[CR84] Weller RO, Boche D, Nicoll JA (2009a) Microvasculature changes and cerebral amyloid angiopathy in Alzheimer’s disease and their potential impact on therapy. Acta Neuropathol 118(1):87–102 19234858 10.1007/s00401-009-0498-z

[CR85] Weller RO, Djuanda E, Yow HY, Carare RO (2009b) Lymphatic drainage of the brain and the pathophysiology of neurological disease. Acta Neuropathol 117:1–14 19002474 10.1007/s00401-008-0457-0

[CR86] Wetjen NM, Heiss JD, Oldfield EH (2008) Time course of syringomyelia resolution following decompression of Chiari malformation Type I. J Neurosurg Pediatr 1(2):118 18352779 10.3171/PED/2008/1/2/118 PMC4294217

[CR87] Winkler EA, Bell RD, Zlokovic BV (2011) Central nervous system pericytes in health and disease. Nat Neurosci 14(11):1398–1405 22030551 10.1038/nn.2946 PMC4020628

[CR88] Xie L, Kang H, Xu Q, Chen MJ, Liao Y, Thiyagarajan M, O’Donnell J, Christensen DJ, Nicholson C, Iliff JJ, Takano T, Deane R, Nedergaard M (2013) Sleep drives metabolite clearance from the adult brain. Science 342(6156):373–377. doi:10.1126/science.1241224 24136970 10.1126/science.1241224 PMC3880190

[CR89] Yurchenco PD, Schittny JC (1990) Molecular architecture of basement membranes. FASEB J 4(6):1577–1590 2180767 10.1096/fasebj.4.6.2180767

[CR90] Zhang E, Inman C, Weller R (1990) Interrelationships of the pia mater and the perivascular (Virchow-Robin) spaces in the human cerebrum. J Anat 170:111 2254158 PMC1257067

[CR91] Zhang E, Richards H, Kida S, Weller R (1992) Directional and compartmentalised drainage of interstitial fluid and cerebrospinal fluid from the rat brain. Acta Neuropathol 83(3):233–239 1373020 10.1007/BF00296784

